# Productive arts engagement at the Tokyo Fuji Art Museum and its health effects on the older Japanese population: results of a randomized controlled trial

**DOI:** 10.3389/fmed.2023.1188780

**Published:** 2023-07-06

**Authors:** Yoko Hayashi, Jacqueline Matskiv, Kevin Galery, Olivier Beauchet

**Affiliations:** ^1^Faculty of Informatics for Arts, Department of Information Expression, Shobi University, Kawagoe, Japan; ^2^Representative Director, Arts Alive, Tokyo, Japan; ^3^Research Centre of the Geriatric University Institute of Montreal, Montreal, QC, Canada; ^4^Departments of Medicine, University of Montreal, Montreal, QC, Canada; ^5^Department of Medicine, Division of Geriatric Medicine, Sir Mortimer B. Davis Jewish General Hospital and Lady Davis Institute for Medical Research, McGill University, Montreal, QC, Canada

**Keywords:** randomized controlled clinical, well-being, quality of life, frailty, Art Museum, productive art engagement

## Abstract

**Background:**

This randomized controlled trial aims to compare changes in mental and physical health in older Japanese community-dwellers who participated in a productive art-based activity at the Tokyo Fuji Art Museum (intervention group) and in their counterparts, who did not participate in the intervention (control group).

**Methods:**

A total of 73 older community-dwellers living in Tokyo participated in a single-blind RCT in two parallel groups (intervention group versus control group). The intervention was 2 h of productive art-based activities per week. The weekly sessions were carried out at the Tokyo Fuji Art Museum over a 12-week period. The control group did not participate in any productive art-based activity over the study period. Well-being, quality of life and frailty were assessed before the first, and after the last, art-based activity. These outcomes were assessed with the same schedule in both groups.

**Results:**

The intervention group saw a significant improvement in their quality of life (*p* < 0.044) and mixed results on their physical health (i.e., decreased frailty status) when compared to the control group. The comparison of changes in frailty scores between M0 and M3 showed improvement in the intervention group (*p* = 0.014), but when adjusted for baseline characteristics by linear regressions, revealed only a trend (*p* = 0.070). No conclusive effect was shown with well-being.

**Interpretation:**

This RCT showed mixed health effects of productive art engagement in older Japanese community-dwellers in Tokyo. Benefits were reported for quality of life and mixed effects were observed for frailty, while no significant effect was found for well-being.

**Clinical Trial Registration**: Ethic committee of Shobi University, Tokyo (Japan), ref. A-2021-1; Clinical Trial Number NCT03679715.

## 1. Introduction

Participating in art-based activities in a museum setting may have health benefits for older adults (1). Both mental and physical health improvements have been associated with productive arts engagement (i.e., doing hands-on activities), such as fine arts-based group activities carried out at the museum, as well as with receptive engagement with art (i.e., attendance of arts-based events and venues) such as guided museum tours (1–3). Visiting museums has also been associated with reduced risk of major neurocognitive disorders and has been shown to contribute to the prevention of cognitive decline (4, 5). Furthermore, an association between art engagement and a lower risk of mortality - particularly among older adults - has also been reported (5, 6).

The health benefits of art engagement have also been confirmed by the World Health Organization’s scoping review on the subject, published at the end of 2019 (3). This review also underscores the need for evidence-based study design, like randomized controlled trials (RCT). Because of their efficacy at reducing confounding factors, RCTs are more likely to generate findings that capture the true effect of an intervention when compared to other research methods (7). Nevertheless, few studies cited in the review used this method (7).

All previous studies on the health benefits of art engagement have been performed in either North America (Canada and the United States) or in Europe, including the United Kingdom (1, 3). Thus, most participants of these studies were Caucasians (3). Ethnicity may influence the health benefits of art engagement, regardless of its type (i.e., productive versus receptive) (8, 9). For instance, there is evidence for lower rates of arts engagement in Black ethnic groups in the United States (9). Currently, there is a lack of data on arts engagement and its health benefits in the Asian population (10). Overall, being Asian does not seem to predict a different rate of engagement with the arts (when compared to white counterparts) after considering factors which may influence this association, like education and income (9). It is clear, however, that this engagement is socially stratified, with people of higher socio-economic status being more likely to engage in the arts (8, 9, 11). Yet both productive and receptive arts engagement have been associated with better holistic wellness and social support in Asian adults aged 50 and above living in Singapore (10).

To date, there have been no published clinical trials involving the older Asian population that track the health benefits of productive arts engagement in a museum setting. Asia is aging at a much faster rate than anywhere else in the world - particularly Japan, which has the highest ratio of people aged 65 and older both in Asia and in the world (12). Because of the potential health benefits of arts engagement in the aging population, there is a need to confirm its effects on the older Japanese population.

Productive arts engagement in a museum setting may improve mental and social health in the older Singaporean population (10). It may also improve physical health in the older North American older population (13). Thus, we hypothesized that productive arts engagement at art museums could improve both the mental and physical health of older Japanese community-dwellers.

This randomized controlled trial aims to compare changes in mental and physical health in older Japanese community-dwellers who participated in a productive art-based activity carried out at the Tokyo Fuji Art Museum (intervention group) and in their counterparts, who did not participate in these art activities (control group).

## 2. Methods

### 2.1. Population

**Figure 1:**
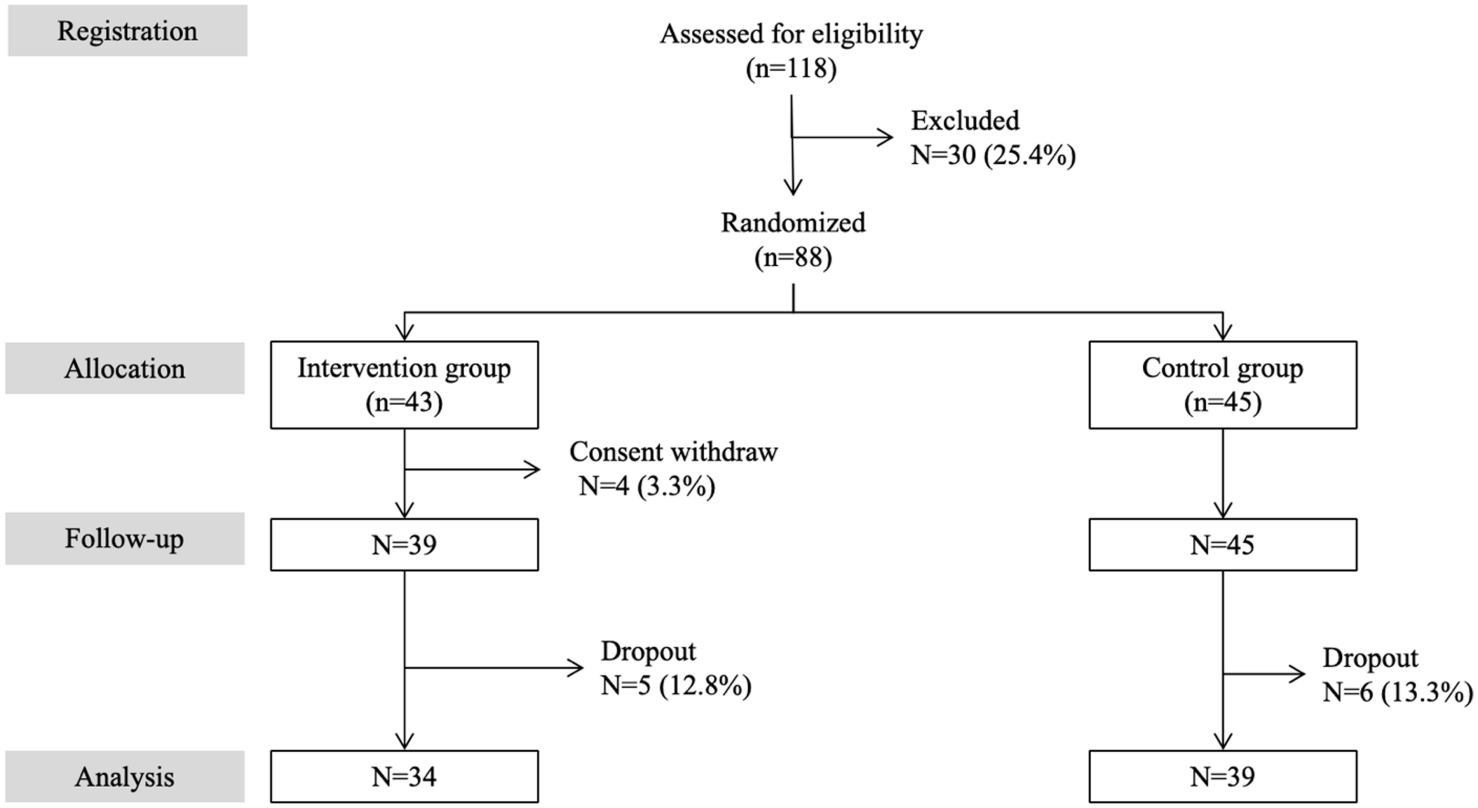
Consort flow diagram detailing selection and follow-up of participants in the RCT.

A total of 73 older community-dwellers, aged 65 and above, who lived in Tokyo and its vicinity (Japan), agreed to participate in this RCT. Arts Alive, which is a non-profit organization in Tokyo, carried out recruitment using various media channels, including direct mailing, college alumni associations, community newspapers, magazine advertisements, and articles in local newspapers, distributing flyers at the museum as well as social networks including Facebook and Twitter. A total of 118 individuals applied to the call. They were then asked to send in signed consent forms to participate in the RCT. We excluded 30 individuals (25.4%), as they declined to participate after being informed of the details of the trial and/or due to an inability to participate due to physical disability. Following the signing of the consent forms and randomization, 4 (3.3%) participants in the intervention group withdrew their consent before the baseline assessment, and 11 participants (9.3%) dropped out over the 3-month intervention period (5 in the intervention group and 6 in the control group). Figure 1 shows the consort flow diagram detailing participant selection and follow-up in the RCT. Participants in the intervention and control groups were recruited and followed over the same period. All recruited participants have no previous experience in arts-based activities. In addition, the control group did not participate in any productive arts-based activity over the study period.

### 2.2. Study design

The study design was an RCT in two parallel groups (intervention versus control). This RCT is registered on the ClinicalTrials.gov website (project number NCT03679715) and followed the RCT guidelines (14). The participants were randomly divided into two groups, intervention (productive arts-based activity) and control (no productive arts-based activity), by block randomization with a block size of 1. The randomization list was generated by an independent assistant coordinator using the N’Query randomization software. The principal investigator and representatives involved in the recruitment and follow-up of participants were blinded to participants’ group assignment. Participants were blinded to assessment results.

### 2.3. Intervention

The current research adopted the standardized 12-week Montreal A-Health participatory art framework with culturally specific modifications that is suitable for the Japan context (i.e., A-Health Japan Framework) (13). The arts intervention designed and executed by Arts Alive in cooperation of the museum consisted of 12 (consecutive) weekly 2-h sessions, which took place at a gallery as well as a lecture hall at the Tokyo Fuji Art Museum over a 3-month period (from May to August 2019). Participants in the intervention group were randomly separated into two smaller groups, so that the same activities were held both in the morning and afternoon on either Saturday or Sunday (due to the availability of the museum space). The 3-month intervention was centered around 3 separate topics, with each topic consisting of 4 consecutive workshops. Once a month, participants also participated in a 30-min-long, dialogue-based art appreciation program called ARTRIP, in which they observed and had discussions on gallery art works in small groups (for a total of three times) with Arts Alive facilitators. The 3 topics of art-making workshops were i) “mural painting with collage” (where participants first made watercolors of plants, animals and other items which constituted a kind of landscape then created a mural with a collage of those watercolors), ii) “from 2 dimensions to 3 dimensions” (which consisted of creating sculpture with paper as well as balsa), and iii) “Japanese mineral pigment painting” (where participants produced their own pigment from mineral rocks and made paintings with them). The workshops and modules were designed to become more challenging as they progressed, so that participants would feel a sense of accomplishment at the end of each session. They were designed by three professional artists together with Arts Alive, while the museum provided the facilities. All workshops were involved interactive, hands-on activities, designed to improve participants’ creativity, observation skills, handicraft techniques, and fine motor skills.

### 2.4. Baseline and follow-up assessments

Assessments were performed before the first workshop (M0) and after the last workshop (M3). Age, sex, polypharmacy (defined as taking 5 or more drugs daily), activities of daily living (ADL) and instrumental activities of daily living (IADL) scales, mood, practice of regular physical activity and history of falls in the past 12 months were assessed (15). In addition, the Centre of Excellence Self-Administered questionnaire (CESAM) was used to assess frailty (16). This validated questionnaire used the deficit accumulation frailty model, which counts health deficits (17, 18). A greater number of deficits indicates a higher frailty state. All CESAM items are close-ended questions: yes = 1 indicates a deficit and no = 0 indicates the absence of a deficit. Two complementary scores are provided by CESAM: 1) a frailty score ranging from 0 (i.e., no deficits) to 18 (i.e., all deficits present) and 2) a frailty stratification into four levels: vigorous (score 0–3), mild frailty (score 4–7), moderate frailty (score 8–12) and severe frailty (score above 12). The Warwick-Edinburgh Mental Well-Being Scale (WEMWBS) questionnaire was used to assess well-being (19). This questionnaire is composed of 14 positively worded items with five response categories. Its score ranges from 14 (i.e., none of the time) to 70 (i.e., all the time). Quality of life was assessed using EuroQol-5D (EQ-5D) (20). This questionnaire is composed of 5 items, with each question’s score ranging from 1 (i.e., no issue) to 5 (i.e., worst issue), yielding a summary score between 0 (i.e., no issue) and 25 (i.e., worst issue). It also includes a visual analogue scale (VAS) representing respondents’ self-perceived health ranging from 0 (i.e., worst health participant can imagine) to 100 (i.e., best health participant can imagine).

### 2.5. Outcomes

The outcomes were (i) mean values of WEMWBS, EQ-5D questionnaires, EQ-5D VAS and CESAM scores, (ii) the distribution of frailty categories (vigorous versus mild, moderate and severe frailty) at M0 and M3, and (iii) changes in outcome mean scores between M0 and M3 using the following formula: [(score M3 – score M0)/(score M3 + score M0)/2] × 100.

### 2.6. Ethical considerations

Recruited participants gave their written informed consent to participate. Ethical approval was obtained for the protocol (Ethics committee of Shobi University, Tokyo (Japan), A-2021-1).

### 2.7. Statistics

Participants’ characteristics were described using means, standard deviations (SD), frequencies and percentages. Unpaired and paired *t*-tests and Chi-squared tests were used for inter and intra-group comparisons. Multiple linear regressions were performed for changes in outcomes between baseline (M0) and the end of the follow-up period (M3), which were significantly different when comparing the intervention and the control group. These linear regressions examined the association of changes in outcome measures (used as dependent variables, separated models for each variable) with the intervention (used as independent variable), adjusted based on the number of workshops performed and the baseline characteristics of participants. *p*-values less than 0.05 were considered statistically significant. All statistics were performed using SPSS (version 26.0; SPSS, Inc., Chicago, IL).

## 3. Results

There was no significant difference among the baseline characteristics of participants, except for mood (Table 1). The participants in the control group were more frequently happy compared to those in the intervention group (*p* = 0.008). No significant change between M0 and M3 in all outcome measures were observed in the control group (Table 2), whereas quality of life (both the EQ-5D questionnaire score and the visual analogue scale) and frailty scores, as well frailty distribution, improved between M0 and M3 (*p* ≤ 0.015) in the intervention group. Comparison of changes between M0 and M3 in outcome measures showed a greater significant improvement in the intervention group compared to the control group for EQ-5D questionnaire scores (*p* = 0.003) and frailty scores (*p* = 0.014) (Table 3). As illustrated in Table 4, linear regressions showed that only the change of the EQ-5D questionnaire score between M0 and M3 was associated with the Tokyo Fuji Art Museum productive arts workshops (coefficient of regression beta = −9,94 with *p*-value = 0.040). Only a trend was observed for the frailty score (coefficient of regression beta = −30.03 with *p*-value = 0.070).

**Table 1:** Baseline characteristics of participants (*n* = 73).

	Participants		
	Control (*n* = 39)	Intervention (*n* = 34)	*p*-value^a^
Age (years), mean ± SD	70.4 ± 4.1	68.7 ± 8.4	0.685
Female, *n* (%)	20 (51.3)	20 (58.8)	0.376
Polypharmacy^b^, *n* (%)	28 (71.8)	25 (73.5)	0.868
ADL score (/6)^c^, mean ± SD	5.9 ± 0.3	5.9 ± 0.3	0.838
IADL score (/4)^d^, mean ± SD	3.1 ± 0.4	3.0 ± 0.3	0.154
Mood happy^e^, *n* (%)	31 (79.5)	17 (50.0)	**0.008**
Practice of physical activity^f^, *n* (%)	34 (87.2)	27 (79.4)	0.372
History of falls in the past 12 months, *n* (%)	17 (17.9)	4 (11.8)	0.461

SD, Standard deviation; ADL, Activity daily living; IADL, Instrumental Activity daily living; CPQ, Computer proficiency questionnaire; *p*-value significant (i.e., <0.05) indicated in bold.

a Comparison based on Mann–Whitney test or chi square, as appropriate.

b Number of drugs taken daily ≥ 5.

c Ranged from 0 (dependent) to 6 (independent).

d Ranged from 0 (non-autonomous) to 4 (autonomous).

e Based on answer to the question “How do you feel today?” with three possible answer including unhappy, happy, neither one nor the other.

f Regular physical activities (walking, bicycle, etc.) at least 1 h per week in the past month.

**Table 2:** Comparisons of mean scores of well-being, quality of life and frailty as well as of frailty categories between control and intervention groups (n = 73).

	Participants						Participants control versus intervention *p*-value	
	Control (*n* = 39)			Intervention (*n* = 34)			M0	M3
	M0	M3	*p*-value^a^	M0	M3	*p*-value^a^		
Warwick-Edinburgh Well-being scale (/70)^b^, mean ± SD	57.4 ± 5.8	58.6 ± 6.4	0.150	57.6 ± 8.3	59.6 ± 7.7	0.105	0.591	0.438
EQ-5D								
Questionnaire score (/25)^c^, mean ± SD	5.7 ± 0.9	5.9 ± 1.1	0.709	3.4 ± 3.3	5.2 ± 0.4	**0.001**	0.691	**0.004**
Visual analogic scale (/100)^d^, mean ± SD	72.5 ± 22.9	80.5 ± 9.4	0.222	69.0 ± 25.5	85.6 ± 10.0	**0.004**	0.525	**0.018**
Frailty^e^								
Score (/18), mean ± SD	3.4 ± 2.0	3.3 ± 1.9	0.503	3.9 ± 2.0	2.5 ± 1.5	**≤0.001**	0.310	**0.047**
Vigorous, *n* (%)	10 (25.6)	13 (33.3)	0.456	10 (29.4)	21 (61.8)	**0.007**	0.719	**0.015**
Mildly frail, *n* (%)	28 (71.8)	24 (61.5)	0.337	23 (67.6)	13 (38.2)	**0.015**	0.700	**0.047**
Moderately frail, *n* (%)	1 (2.6)	2 (5.1)	0.556	1 (2.9)	0 (0)	0.314	0.922	0.181

SD, Standard deviation; EQ-5D, EuroQol 5D; M, Month; M0, baseline assessment before intervention; M3, assessment at the end of the 3-month period of intervention; a ‘mild’ frail score ranges from 4 to 8; a ‘moderate’ frail score ranges from 9 to 14; a ‘severe’ frail score is ≥ 15; *p*-value significant (i.e., <0.05) indicated in bold.

a Comparisons based Wilcoxon or chi squares, as appropriate.

b Ranged from 14 (i.e., none of the time) to 70 (i.e., all the time).

c score ranged from 0 (no problem) to 25 (unable to do).

d scored ranges from 0 (the worst health condition) to 100 (the best health condition).

e Mean score calculated from computerized self-administered questionnaire composed of 20 questions providing a score ranged from 0 (vigorous) to 18 (severe frail) and three categories (a ‘vigorous’ score ranged from 0 to 3).

**Table 3:** Comparisons of score variations of well-being, quality of life and frailty between the baseline assessment and the end of the 3-month period of follow-up in control (*n* = 39) and intervention (*n* = 34) groups.

Variations^a^ of scores between the baseline assessment and the end of the 3-month follow-up, mean ± SD (%)	Control (*n* = 39)	Intervention (*n* = 34)	*p*-value^b^
Warwick-Edinburgh Well-being scale^c^	2.0 ± 7.9	3.7 ± 14.2	0.699
EQ-5D			
Questionnaire score^d^	1.7 ± 18.1	−13.3 ± 25.9	**0.003**
Visual analogic scale^e^	16.4 ± 45.3	27.8 ± 51.7	0.174
Frailty score^f^	−6.1 ± 60.2	−47.7 ± 68.3	**0.014**

SD, Standard deviation; EQ-5D, EuroQol 5D; *p*-value significant (i.e., <0.05) indicated in bold.

a Calculated with the formula: Difference between the baseline assessment (M0) and the end of the 3-month period of follow-up (M3): ((score after – score before)/((score after + score before)/2)) × 100.

b Based on Mann–Whitney test.

c Ranged from 14 (i.e., none of the time) to 70 (i.e., all the time).

d Score ranged from 0 (no problem) to 25 (unable to do).

e Scored ranges from 0 (the worst health condition) to 100 (the best health condition).

f Mean score calculated from computerized self-administered questionnaire composed of 20 questions providing a score ranged from 0 (vigorous) to 18 (severe frail).

**Table 4:** Multiple linear regressions showing the association of score variations of quality of life and frailty between the baseline assessment and the end of the 3-month follow-up (used as dependent variables, separated models for each variable) with the intervention (used as an independent variable), adjusted according to the number of workshops performed and the baseline characteristics of participants (*n* = 73).

Variations^a^ of scores between the baseline assessment and the end of the 3-month period of follow-up	Intervention		
	*ß*	[95%CI]	*p*-value
EQ-5D Questionnaire score^b^	−9.94	[−19.59;-0.29]	**0.044**
Frailty score^c^	−30.03	[−62.57;2.51]	0.070

EQ-5D, EuroQuol 5D; ß, coefficient of regression beta; CI, Confidence interval; *p*-value significant (i.e., <0.05) indicated in bold.

a Calculated with the formula: Difference between the baseline assessment (M0) and the end of the 3-month period of follow-up (M3): (score after – score before)/(score after + score before)/2) ×100.

b Score ranged from 0 (no problem) to 25 (unable to do).

c Mean score calculated from computerized self-administered questionnaire composed of 20 questions providing a score ranged from 0 (vigorous) to 18 (severely frail).

## 4. Discussion

The findings showed mixed health effects of museum-based, productive arts engagement by older Japanese community-dwellers living in Tokyo. Benefits were reported for quality of life, while mixed results were observed for frailty and non-conclusive effect was found for well-being.

Both improvement of quality of life and mental health benefits are the most commonly reported positive effects of arts engagement, regardless of the type (i.e., productive versus receptive) and setting (3). Arts engagement is a multimodal intervention involving imagination, sensory activation, cognition and emotion (1–3, 14, 20–23). This multimodal stimulation produces psychological effects including happiness, enhanced self-efficacy, self-esteem, and positive emotions (1–3). All these psychological effects may improve quality of life because they influence the individual’s perception of their life (19). Quality of life is defined by the World Health Organization as the “individuals’ perception of their position in life in the context of the culture and value systems in which they live, and in relation to their goals, expectations, standards and concerns” (24).

In our study, we reported an improvement in quality of life, but no effect on well-being was observed. Well-being and quality of life both refer to a positive and subjective sense of health (3, 24, 25). And while the link between the two is undeniable, they refer to two separate, yet complementary domains of wellness (25). Quality of life is located in the objective realm, at the intersection of individual needs and external resources. Well-being, on the other hand, captures one’s ability to take advantage of available resources and experience satisfaction, which places well-being in the subjective realm (25). In our study, the mood status, respectively, reported by intervention and control groups differed at baseline. A happy mood was less prevalent in the intervention group compared to the control group, which may influence their receptiveness to arts engagement. Furthermore, we used the WEMWBS questionnaire, which is composed of 14 positively-worded items, to assess well-being (19). Thus, it may be suggested that participants with a positive mood tended to score higher compared to those with a lower mood. In addition, the WEMWBS has been developed and validated in United Kingdom in Caucasian population (19). A cultural effect in the Japanese participants of our RCT may be evoked to explain the absence of significant improvement of WEMWBS score. A cultural effect encompasses the ways in which culture shapes and molds people’s behaviors, attitudes, values, customs, and social interactions. Its effect may influence one’s sense of happiness (i.e., in what circumstances one feels happy). Thus, WEMWBS may be not adapted to assess well-being in the Japanese population.

We observed mixed results for physical health. The comparison of changes in frailty scores between baseline and the end of the follow-up period showed that the level of frailty decreased significantly in the intervention group when compared to the control group. However, we reported only a trend of this improvement when adjusting for baseline characteristics. It has been reported in previous clinical trials that arts-based activities practiced at the museum improved the frailty state of older community-dwellers (13, 26–28). Our results are consistent with these previous results. We showed that there was a significant decrease in CESAM scores in the intervention group compared to the control group at M3, indicating a physical health improvement. Moreover, the change in CESAM scores between M0 and M3 was greater in the intervention group compared to the control group. It has been reported that frailty may be prevented or even reversed, especially at its onset (29). Older individuals with mild frailty seem to benefit the most from interventions that can promote health and prevent frailty from worsening. The result of our RCT seems to confirm this statement. However, the non-conclusive results underscored by the linear regression and its adjustment by the baseline characteristics suggested that the association is weak. One explanation of this result may be related to the low number of participants in the RCT and, thus, a lack of power to show a significant association. At a health policy level, as exemplified by the English Alliance of Museums for Health and Well-being (30), the results of this RCT highlight that art museums may become important agents of health promotion among the older population.

The present RCT has a number of limitations that should be taken into account. First, it was carried out only at the Tokyo Fuji Art Museum. Second, the control group may have been exposed to various activities that influenced the outcomes over the 3-month period of the RCT. We suggest that this effect was limited by our control methods. Third, although we controlled for characteristics that may impact the intervention, residual confounding might still be present. For instance, analyses were adjusted for the covariates measured at baseline, but not for their change from baseline to follow-up. As confounding can impact both the magnitude and direction of associations, it is difficult to speculate on the possible impact of residual confounders on the associations found in the present study. In addition, we did not adjust according to education level and the socio-economic condition of participants, which are two covariates which may influence the examined association. Fourth, data were collected through self-assessments, specifically self-reported questionnaires. The main disadvantage of these questionnaires is the subjectivity of responses, which may lead to biases, such as the desirability or response bias, resulting in inaccurate answers (31). Fifth, the low number of participants and the short period of intervention and follow-up of 3 months are limitations. Non-conclusive results may be explained by a lack of power or a too-short intervention period. All these limitations underscore the need to reproduce the RCT with more participants, a longer follow-up period, an analysis of the cost effectiveness of the productive art activities and a mixed design combining quantitative and qualitative data.

In conclusion, this RCT showed mixed effects of productive arts engagement in older Japanese community-dwellers residing in Tokyo. Benefits for quality of life and frailty were reported but no significant effect for well-being was demonstrated. Further research needs to be performed to determine the possible benefits of productive, museum-based arts engagement in Asian populations.

## Data availability statement

The original contributions presented in the study are included in the article/supplementary material, further inquiries can be directed to the corresponding author.

## Ethics statement

The studies involving human participants were reviewed and approved by Ethics committee of Shobi University, Tokyo (Japan). The patients/participants provided their written informed consent to participate in this study.

## Author contributions

OB and YH conceived and designed the experiments. YH performed the experiments. OB and KG analyzed and interpreted the data. YH and KG contributed reagents, materials, analysis tools or data. OB, KG, and YH writing of the manuscript. JM revision of manuscript. All authors contributed to the article and approved the submitted version.

## Conflict of interest

The authors declare that the research was conducted in the absence of any commercial or financial relationships that could be construed as a potential conflict of interest.

## Publisher’s note

All claims expressed in this article are solely those of the authors and do not necessarily represent those of their affiliated organizations, or those of the publisher, the editors and the reviewers. Any product that may be evaluated in this article, or claim that may be made by its manufacturer, is not guaranteed or endorsed by the publisher.
